# Acute Non-Obstructive Bilateral Pyelonephritis With Acute Kidney Injury Requiring Hemodialysis

**DOI:** 10.7759/cureus.26746

**Published:** 2022-07-11

**Authors:** Arjun Mainali, Samaj Adhikari, Tutul Chowdhury, Nicole Gousy, Navodita Uprety, Amita Arora, Carlos J Palencia

**Affiliations:** 1 Internal Medicine, Interfaith Medical Center, Brooklyn, USA; 2 Medicine, American University of Antigua, New York, USA; 3 Medicine, Interfaith Medical Center, Brooklyn, USA; 4 Internal Medicine, Nephrology, Endocrinology, Rheumatology, University of Carabobo, Valencia, VEN

**Keywords:** acute renal failure and hemodialysis in icu, bilateral pyelonephritis, non-obstructive pyelonephritis, acute pyelonephritis, acute kidney injury

## Abstract

Acute pyelonephritis (APN) is considered a rare cause of acute kidney injury (AKI), especially when no anatomical abnormalities or predisposing factors are identified. Additionally, non-obstructive pyelonephritis is a very infrequent cause of rapidly progressive acute kidney injury. Herein, we present a rare case of a 55-year-old female patient who was diagnosed with acute non-obstructive pyelonephritis leading to AKI eventually requiring hemodialysis. The patient eventually recovered with the administration of intravenous antibiotics with a significant recovery of renal function.

## Introduction

A significant number of patients with acute pyelonephritis (APN) require hospitalization each year in the USA [1]. Acute pyelonephritis without any anatomical abnormality is a rare clinical entity. Additionally, acute non-obstructive pyelonephritis with severe acute kidney injury (AKI) requiring hemodialysis is a clinical challenge infrequently encountered. The major mechanisms for acute renal failure secondary to pyelonephritis are mediated by cytokine-mediated injury or acute tubular injury due to hypotension secondary to shock. Prompt management of pyelonephritis, specifically among immunocompromised patients, is essential to prevent shock and permanent damage to the kidneys. Acute bilateral non-obstructive pyelonephritis among diabetic patients has been previously reported [2,3]. Here, we present a rare case of acute non-obstructive bilateral pyelonephritis with acute renal failure in a patient with HIV requiring hemodialysis.

## Case presentation

A 55-year-old female with a past medical history of bronchial asthma, hypertension, and HIV on highly active antiretroviral therapy (HAART) therapy presented to the emergency department (ED) with complaints of constant severe diffuse abdominal pain associated with nausea and three episodes of non-bilious, non-projectile vomiting. She reported burning with urination but denied urgency or frequency of urination. She also denied having a fever during this episode. Other reviews of systems were unremarkable. She was admitted one month ago for approximately four days to undergo a laparoscopic cholecystectomy. She has a history of a penicillin allergy resulting in a pruritic rash after administration. She denied smoking, using alcohol, and using illicit drugs. Family history is not significant for any connective tissue disorder.

On physical examination, she was in mild distress due to pain. Triage vitals showed blood pressure at 78/56 mmHg, heart rate at 94 beats/min, temperature at 36.9, respiratory rate at 18 breaths/min, and oxygen saturation status of 97% in room air. An abdominal exam revealed a soft abdomen with mild diffuse generalized abdominal tenderness and bilateral renal angle tenderness; however, no guarding or rigidity was appreciated. The respiratory exam showed bilateral vesicular breath sounds with no added sounds. On the cardiovascular exam, S1 and S2 sounds were appreciated at a regular rate and rhythm without murmurs, rubs, or gallops. All other physical exams, including the central nervous system exam, were normal. Appropriate labs done at the time of admission are shown in Tables 1-3.

**Table 1:** Results of the patient's complete blood count and metabolic panel in addition to inflammatory markers, coagulation profile, viral load, absolute CD4 helper cell count, and Hepatitis B and C serology WBC: white blood cell count; Hg: hemoglobin; BUN: blood urea nitrogen; AST: aspartate aminotransferase; ALT: alanine aminotransferase; ALP: alkaline phosphatase; iPTH: intact parathyroid hormone; BNP: brain natriuretic peptide; PT: prothrombin time, INR: international normalized ratio; PTT: partial thromboplastin time, HbsAG: hepatitis B surface antigen

Test	Ref range and units	Values
WBC	4.5–11.0 10 × 3/µL	7.8
Neutrophil %	40.0–70.0%	88
Lymphocytes %	22.0–48.0%	6.6
Monocytes %	2.0–14.0%	1.2
Eosinophil %	0.5–5.0%	4.1
Basophil %	0.0–2.0%	0.1
Hb	11.0–15.0 g/dL	11.6
BUN	7.0–18.7 mg/dL	73.3
Creatinine	0.57–1.11 mg/dL	8.68
Na	136–145 mmol/L	136
K	3.5–5.1 mmol/L	4.4
CO2	22–29 mmol/L	15
Total Bilirubin	0.2–1.2 mg/dL	0.5
ALT	10–55 U/L	39
AST	5–34 U/L	61
ALP	40–150 U/L	139
Albumin	3.5–5.2 g/dL	3.5
Phosphorous	2.3–4.7 mg/dl	3
iPTH	7.5–53.5 pg/ml	158
Ca	8.4–10.2 mg/dL	7.8
Phosphorus	2.3–4.7 mg/dL	6.5
BNP	10–100 pg/ml	1602
Lactate	0.50–1.90 mmol/L	3.79
PT	9.8–13.4 sec	15.1
INR	0.85–1.15	1.25
PTT	24.9–35.9 sec	39.4
D-dimer	≤500 ng/ml	868
Vitamin D 25 hydroxy	30–100 ng/ml	35.4
Procalcitonin	0.00–0.08 ng/ml	1.16
C-Reactive protein (CRP)	0.5–1 mg/dl	8.50
Erythrocyte sedimentation rate (ESR)	0–22 mm/hr	>120
HIV-1 RNA PCR	Copies/ml	<40
Absolute CD4 helper	359–1519 /uL	462
HbsAG	Negative	Negative
Hepatitis C	Negative	Negative

**Table 2:** Patient’s urinalysis and microscopic with urinary electrolytes

Test	Ref range and units	Values
Color	Light yellow	Yellow
Clarity	clear	Clear
Specific gravity	1.005–1.030	>1.015
pH	5.0-8.0	8.5
Glucose	0 mg/dL	Negative
Protein	0 mg/dL	100
Blood	Negative	Moderate
Nitrite	Negative	Negative
Leukocytes	Negative	Negative
WBC	0–5/HPF	5-10
RBC	0–4/HPF	>100
Bacteria	None/HPF	Many
Potassium urine random	3.5–5.1 mmol/L	28.3
Chloride urine	mmol/L	53.08
Sodium urine random	mmol/L	68.9

**Table 3:** Patient’s Covid-19 and influenza test

Test	Ref range and units	Values
SARS-Cov-2 PCR	Not detected	Not detected
Influenza A, NAA	Not detected	Not detected
Influenza B, NAA	Not detected	Not detected

**Figure 1:**
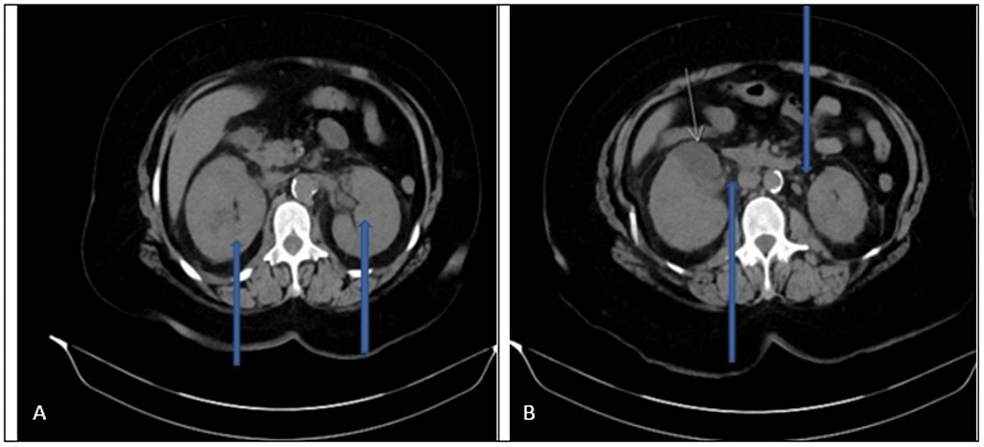
CT abdomen without contrast showing bilateral enlarged kidneys as shown by the blue arrows (A). Additionally bilateral perinephric fat stranding (blue arrows) with wedge-shaped low density in the anterior pole of the right kidney as shown by the yellow arrow (B) consistent with radiologic findings of bilateral acute pyelonephritis.

The patient's baseline urea and creatinine one month ago were 14.3 mg/dl and 1.08 mg/dl, respectively. Based on her clinical presentation and physical findings of low blood pressure, tachycardia, bilateral renal angle tenderness, and urinalysis showing typical features of urinary tract infection, sepsis likely from acute bilateral pyelonephritis was considered. Urine toxicology was negative, and urinalysis did not show any glomerular casts. She was started on broad-spectrum antibiotics, aztreonam (due to penicillin allergy reported by the patient), and vancomycin. She received an initial fluid bolus of 30 ml/kg over three hours, and her blood pressure improved with the normalization of lactic acid. Intravenous fluid with 0.9% normal saline was continued for hydration of the kidney. A foley catheter was inserted for strict urine input and output charting with an initial collection of only 100 ml of urine after the insertion. As she was having diffuse abdominal pain, a non-contrast CT of the abdomen was performed to rule out other causes of acute abdomen and obstructive renal pathology. The CT scan showed bilateral pyelonephritis, as described in Figure 1, with an absence of obstruction in the urinary tract. An echocardiogram was done in view of an elevated BNP, which showed left ventricular systolic dysfunction with an ejection fraction of 40-45%.

An autoimmune workup was also done to rule out other causes of acute renal failure as described in Table 4, all of which were negative.

**Table 4:** Patient’s immunology results ANA: antinuclear antibodies, RNP: ribonucleoprotein, ANCA: antineutrophil cytoplasmic antibodies

Test	Ref range and units	Values
ANA	Negative	Negative
Anti-DNA (DS) Ab Qn	0-9 IU/mL	<1
RNP antibodies	0.0-0.9 AI	<0.2
Smith antibodies	0.0-0.9 AI	<0.2
Antiscleroderma-70 Abs	0.0-0.9 AI	<0.2
Sjogren’s anti-SS-A	0.0-0.9 AI	<0.2
Sjogren’s anti-SS-B	0.0-0.9 AI	<0.2
Antiribosomal P antibodies	0.0-0.9 AI	<0.2
Complement C3, serum	82-167 mg/dl	109
Complement C4, seum	12-38 mg/dl	38
Cytoplasmic (C-ANCA)	Neg:<1:20 titer	<1:20
Perinuclear (P-ANCA)	Neg:<1:20 titer	<1:20

**Figure 2:**
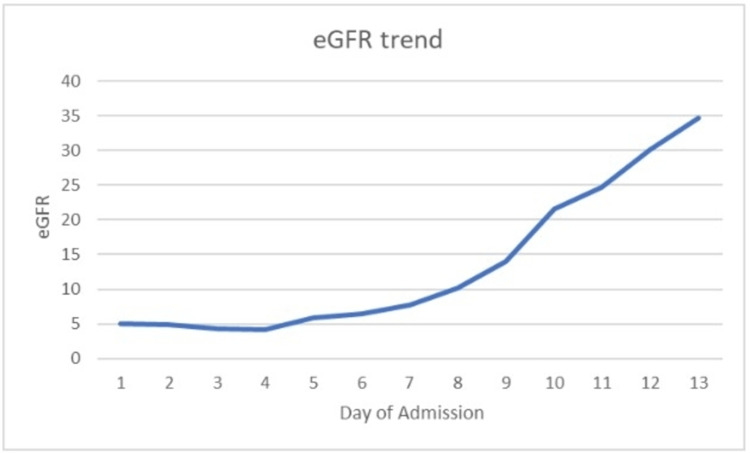
Patient's estimated glomerular filtration rate trended over course of admission

The patient’s urine output did not improve even after fluid resuscitation, and her BUN/creatinine trended up with the decrease in estimated glomerular filtration rate (eGFR), as shown in Figure 2.

Her blood and urine cultures showed *Klebsiella pneumonia* which was pansensitive, and later antibiotics were de-escalated to aztreonam only. The nephrology team was consulted, and on the fourth day of admission, she underwent one session of hemodialysis. Her urine output and kidney functions started improving without further dialysis. She improved clinically, and the foley was discontinued. In view of negative autoimmune work up with clinical features and imaging highly suggestive of acute bilateral pyelonephritis, a renal biopsy was not considered. The patient’s antibiotic was changed to oral levofloxacin after seven days of treatment with aztreonam to complete 14 days of treatment.

## Discussion

Acute kidney injury is defined as an acute decline in kidney function due to an insult or injury resulting in a loss of renal function [3]. Acute kidney injury can be further classified as either prerenal, intrarenal, or post-renal [4]. While there are many common causes of acute kidney injury, pyelonephritis, as the root cause of acute kidney injury, is rarely seen in the adult population. Generally, pyelonephritis-induced acute kidney injury has been more commonly reported in the pediatric population due to anatomic variations. Contrastingly, non-obstructive pyelonephritis as a cause of acute kidney injury has an incidence of approximately 2-3%, with some literature reporting incidences as low as 0.7% [4-7]. Hence, the diagnosis of pyelonephritis leading to acute kidney injury is exceedingly rare and infrequently reported in the literature [4].

Pyelonephritis is most commonly reported in women [1], but men have a higher mortality rate [8]. Common presiding factors for pyelonephritis include prolonged use of indwelling catheters, renal stones, pregnancy, and chronic use of medications such as non-steroidal analgesics, or immunosuppressive agents [9]. It is possible that this patient’s HIV status may have contributed to the development of AKI secondary to pyelonephritis. Regardless of the predisposing factors, pyelonephritis can induce acute renal injury by disrupting tubular function via interstitial inflammation and edema, with eventual tubular obstruction with cellular debris and increased vasoconstriction of the renal microvasculature [8]. This creates a cycle of increased inflammation and edema, leading to more tubular obstruction, leading to significant renal dysfunction, which can necessitate the use of hemodialysis, as seen in this patient.

Common complications of pyelonephritis can include the formation of a renal abscess, permanent parenchymal destruction that can lead to scarring, and a significant and potentially permanent loss or reduction in renal function [3]. The diagnosis of pyelonephritis involves performing a urinalysis with a subsequent urine culture. Typically, imaging is not a routine step in the diagnosis of pyelonephritis, but it can be utilized when there is no improvement seen within 72 hours of initiating appropriate antibiotic therapy. Antibiotic susceptibility testing is commonly used when determining the sensitivity of the causative organism, and earlier utilization of an appropriate antibiotic generally has a better patient outcome [9].

While the incidence of bilateral non-obstructive pyelonephritis is rare, studies have shown that the risk of developing acute kidney injury is greater in those with bilateral renal involvement compared to unilateral pyelonephritis [3,4,10]. Clinically, unilateral and bilateral pyelonephritis present similarly with systemic symptoms including nausea, fever, increased urinary frequency and urgency, and flank pain. However, it has been noted that those with bilateral pyelonephritis have a more complicated disease progression and a poorer outcome compared to those with unilateral pyelonephritis. This is comparable to this patient who required hemodialysis before eventually regaining her renal function.

## Conclusions

Acute non-obstructive bilateral pyelonephritis can lead to septic shock and can even cause permanent renal damage. Our patient with HIV status makes the case report unique. Clinicians should remain vigilant in prompt management among patients with baseline immunocompromised status with early administration of antibiotics for complete recovery of renal function.
